# Treinamento de Não-Cardiologistas pode Melhorar os Resultados do Tratamento de Infarto Agudo do Miocárdio com Supra de ST

**DOI:** 10.36660/abc.20200180

**Published:** 2021-08-02

**Authors:** Luiz Antonio Machado Cesar, Antonio Padua Mansur, Rui Fernando Ramos, Carlos Magalhães, João Fernando Monteiro Ferreira, Naide Aparecida de Oliveira, Amaury Zatorre Amaral, Antonio Célio Camargo Moreno

**Affiliations:** 1 Instituto do Coração Hospital das Clínicas FMUSP São Paulo SP Brasil InCor - Instituto do Coração do Hospital das Clínicas da FMUSP, São Paulo, SP - Brasil; 2 Instituto Dante Pazzanese de Cardiologia São Paulo SP Brasil Instituto Dante Pazzanese de Cardiologia, São Paulo, SP - Brasil; 3 Universidade Nove de Julho São Paulo SP Brasil Universidade Nove de Julho, São Paulo, SP - Brasil; 4 Secretaria da Saúde do Estado de São Paulo São Paulo SP Brasil Secretaria da Saúde do Estado de São Paulo, São Paulo, SP - Brasil; 5 Secretaria Municipal da Saúde São Paulo SP Brasil Secretaria Municipal da Saúde, São Paulo, SP - Brasil; 6 Secretaria de Gestão Pública São Paulo SP Brasil Secretaria de Gestão Pública, São Paulo, SP - Brasil

**Keywords:** Síndrome Coronária Aguda, Infarto do Miocárdio/tratamento medicamentoso, Capacitação, Ensino, Epidemiologia, Mortalidade, Serviços de Emergência

## Abstract

**Fundamento:**

De acordo com a Organização Mundial da Saúde, países emergentes terão um crescimento considerável no número de ataques cardíacos e mortes relacionadas. Um dos principais problemas médicos no Brasil é a mortalidade causada por infarto agudo do miocárdio com supra de ST (IAMCSST). A Sociedade de Cardiologia do Estado de São Paulo nunca treinou não-cardiologistas para atendimentos de emergência. Os pacientes normalmente buscam ajuda em prontos-socorros, em vez de chamar a ambulância.

**Objetivo:**

Nosso objetivo foi reduzir as taxas de mortalidade hospitalar causada por infarto agudo do miocárdio ao treinar profissionais da emergência na cidade de São Paulo.

**Métodos:**

Utilizamos um programa de treinamento para as equipes de cinco hospitais com > 100 pacientes internados com IAMCSST por ano, e pelo menos 15% de mortes hospitalares relacionadas ao IAMCSST. Realizamos treinamentos online, organizamos de dois a quatro eventos para até 400 participantes, fizemos folders e panfletos informativos. A análise estatística utilizou o teste para comparação de duas proporções, com p <0,05.

**Resultados:**

Quase 200 médicos e 350 enfermeiros participaram de pelo menos um treinamento de maio de 2010 até dezembro de 2013. Inicialmente, muitos médicos da emergência não reconheciam um infarto agudo do miocárdio no eletrocardiograma, mas a tele-ecocardiografia é usada em alguns departamentos da emergência para determinar o diagnóstico. A taxa de mortalidade nos cinco hospitais caiu de 25,6%, em 2009, para 18,2%, em 2010 (p=0,005). Depois da conclusão do período de treinamento, as mortes relacionadas ao IAMCSST em todos os hospitais públicos de São Paulo diminuíram de 14,31%, em 2009, para 11,25%, em 2014 (p<0,0001).

**Conclusão:**

Mesmo programas simplificados de treinamento de pessoal da emergência pode reduzir muito as taxas de morte por infarto agudo do miocárdio em países em desenvolvimento.

## Introdução

Doenças cardiovasculares continuam sendo a principal causa de morte em muitos países. De acordo com a Organização Mundial da Saúde, países emergentes apresentarão um grande crescimento no número de ataques cardíacos e, consequentemente, no número de mortes.^1^ Em 2010, havia 200 milhões de habitantes no Brasil, e uma incidência estimada de 116 ataques cardíacos por 100.000 pessoas,^2^ em comparação a 294 por 100.000 nos Estados Unidos.^3^ O principal problema médico no Brasil é a mortalidade causada pelo infarto agudo do miocárdio com supra de ST (IAMCSST), o que já não é o caso em países desenvolvidos.^4^ A maior cidade do Brasil, a região metropolitana de São Paulo, tem aproximadamente 18 milhões de habitantes, a maioria dos quais depende do sistema público de saúde da cidade. As autoridades públicas estimam que ≥ 70% da população utiliza esses serviços, desde a atenção primária até tratamentos especializados. A taxa de mortalidade hospitalar da cidade causada pelo IAMCSST em 2009 foi de 14,1%,^5^ quase duas vezes a porcentagem encontrada em países desenvolvidos. Naquela época, o tratamento disponível para IAMCSST na maioria dos hospitais de São Paulo era a terapia trombolítica com uso da estreptoquinase. Hoje, o sistema público de saúde tem 46 hospitais gerais, 139 unidades de emergência para atenção básica e 400 ambulatórios. Dos 46 hospitais gerais, somente seis podem realizar a intervenção coronária percutânea (ICP) primária, e não há um sistema organizado para transferir pacientes para esses hospitais para realizar a ICP, nem para submeter os pacientes à angiografia coronária imediatamente após a trombólise. A Sociedade de Cardiologia do Estado de São Paulo, que faz parte da Sociedade Brasileira de Cardiologia, pensou em aprimorar o cuidado dirigido a esses pacientes. A Sociedade tem treinado cardiologistas há muitos anos, mas os cardiologistas não são os profissionais que estão nos prontos-socorros; lá ficam os médicos internos e especialistas como ginecologistas e ortopedistas.

Treinamentos, reforço dos treinamentos e revisão são ferramentas importantes para qualquer atualização na área médica, mas talvez não sejam necessários para conteúdos muito básicos. Outras iniciativas semelhantes trouxeram resultados esclarecedores em outros países.^6 - 12^ Considerando a relativa simplicidade do diagnóstico e do tratamento do IAMCSST, não está claro se o treinamento básico pode, de fato, reduzir as taxas de mortalidade. Dentre os hospitais públicos de São Paulo, somente aqueles com unidades especializadas de cardiologia podem realizar a ICP, e a mortalidade por IAMCSST é aceitável (6 a 7%) nesses hospitais. Alguns dos 24 hospitais que não oferecem tratamentos na área da cardiologia intervencionista tinham uma taxa de morte relacionada ao IAMCSST >15% em 2009, de acordo com dados da Secretaria de Saúde do Estado de São Paulo.^13^ A Sociedade de Cardiologia do Estado de São Paulo treina cardiologistas, inclusive para o diagnóstico e tratamento da IAMCSST, desde 1976, mas nunca treinou profissionais não-cardiologistas que atuam na emergência. Os moradores de São Paulo não costumam ligar para o 193 (similar ao 911, nos Estados Unidos) quando sentem dor aguda no peito; preferem ir ao pronto-socorro, unidades básicas de saúde e hospitais gerais da cidade. Assim, nosso objetivo foi o de reduzir a taxa de mortalidade hospitalar relacionada ao IAMCSST oferecendo um programa de treinamento para os profissionais da emergência (médicos, enfermeiros e outros) na cidade de São Paulo.

## Métodos

Depois de três reuniões com os chefes de unidades de emergência e departamentos de pronto-atendimento de hospitais, a Sociedade de Cardiologia, juntamente com a Secretaria de Saúde do Estado de São Paulo e a Secretaria de Saúde da cidade de São Paulo, desenvolveram um programa de treinamento.

Nós pré-estabelecemos um primeiro objetivo: ensinar os profissionais dos cinco hospitais que tiveram mais de 100 pacientes com IAMCSST em um ano e taxa de mortalidade hospitalar relacionada ao IAMCSST ≥15%. Para esses cinco hospitais, realizamos treinamentos presenciais aos sábados. Durante os encontros, observamos que muitos participantes tinham medo de iniciar a terapia trombolítica.

Além do treinamento presencial, usamos ferramentas virtuais para os treinamentos online, fizemos reuniões de atualização, como os simpósios para até 400 participantes de duas a quatro vezes por ano, criamos informativos virtuais e panfletos. As reuniões presenciais e os simpósios incluíram um programa de treinamento de quatro horas, com três seminários abordando o diagnóstico diferencial de dor no peito, dor torácica e diagnóstico e tratamento do infarto agudo do miocárdio (IAM) no departamento de emergência. Depois de cada treinamento, havia uma sessão de perguntas e respostas. Médicos, enfermeiros e outros profissionais da emergência foram convidados a participar.

O principal objetivo foi avaliar os efeitos do treinamento nas taxas de mortalidade hospitalar relacionadas ao IAMCSST. Durante os três anos, a estreptoquinase foi substituída pela tenecteplase em alguns hospitais. Depois do tratamento com trombolíticos, aspirina, clopidogrel e enoxaparina, os pacientes foram transferidos para um hospital terciário capaz de fornecer tratamentos de cardiologia intervencionista e cirurgia. A incidência de IAM e sua taxa de mortalidade associada foi atualizada a cada semestre pela Secretaria de Saúde do Estado de São Paulo. Esta informação veio de hospitais públicos, que preencheram formulários sobre internação, alta e taxas de mortalidade hospitalar.

Para avaliar a influência tanto do programa de treinamento como da estratégia da tecnecteplase seguida de transferência para um hospital de referência nas taxas de mortalidade, monitoramos especificamente um hospital que introduziu a tecnecteplase e avaliou as taxas de mortalidade quatro meses após o treinamento; depois, avaliou o início da estratégia da tecnecteplase ao longo dos anos, depois de 2013 até 2015. Além disso, avaliamos os mesmos dados de outros cinco hospitais com taxa de mortalidade associada ao IAMCSST ≥15% como controle. Um Conselho de Ética Institucional aprovou o estudo, cujos dados foram coletados da Secretaria de Saúde do Estado de São Paulo, Brasil.

### Análise Estatística

Obtivemos os dados para este estudo da Secretaria de Saúde da cidade de São Paulo. A base de dados continha informações de cada unidade de saúde onde um paciente tenha sido admitido, gerando uma autorização para internação. Utilizamos o número de mortes (N) e as taxas de mortalidade (%) para realizar o teste para comparação de duas proporções, usando o software Primer of Biostatistics ^®^, versão 4.02.9.^14^ Valor de p foi considerado significativo quando < 0,05.

## Resultados

**Figura 1:**
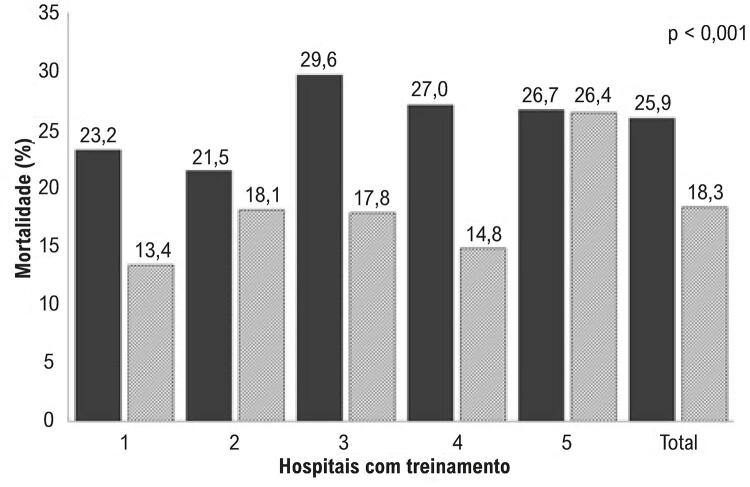
– *Taxa de mortalidade (%) nos primeiros cinco hospitais que participaram do programa de treinamento. Comparação entre 2008-2009 e 2010 (valor de p para comparação). Colunas: cinza escuro=antes; e cinza claro: pós-treinamento. Os números acima das colunas mostram a exata porcentagem da taxa de morte em cada hospital.*

**Figura 2:**
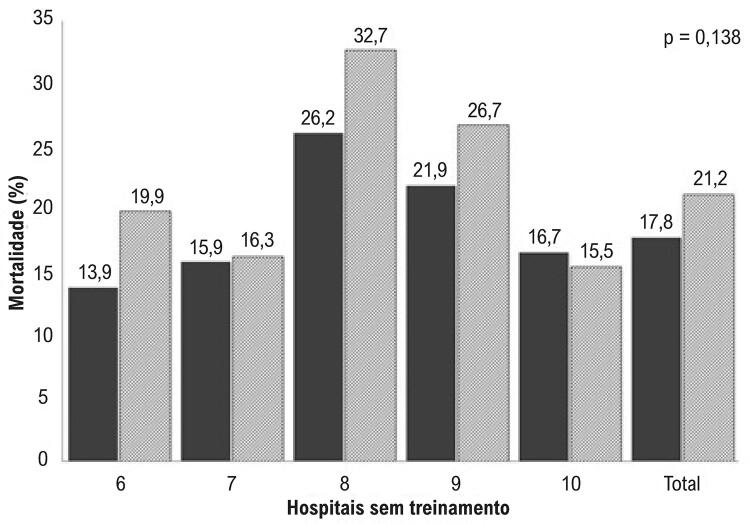
– *Taxa de mortalidade (%) nos cinco hospitais que não receberam o treinamento. Comparação entre 2008-2009 e 2010 (valor de p para comparação). Colunas: cinza escuro=antes; e cinza claro: pós. Os números acima das colunas mostram a exata porcentagem da taxa de morte em cada hospital.*

Oferecemos treinamento presencial para profissionais da emergência de cinco hospitais, e estendemos o treinamento a três outros hospitais em 2010. Os mesmos participantes passaram por treinamento de reciclagem virtuais e simpósios de atualização. Doze unidades de emergência também treinaram suas equipes. Muitos outros hospitais treinaram seu pessoal de emergência e terapia intensiva de 2011 a 2013. No total, aproximadamente 200 médicos e 350 enfermeiros participaram de pelo menos uma sessão de treinamento de maio de 2010 até dezembro de 2013. Observamos que muitos médicos da emergência não conseguiram identificar um IAM no eletrocardiograma. Quase 50 departamentos de emergência usaram a tele-eletrocardiografia, que causa um atraso de cinco minutos no diagnóstico. Mesmo naqueles departamentos, a equipe de emergência costumava ter medo de iniciar a terapia trombolítica. As taxas de mortalidade associadas ao IAMCSST nos cinco hospitais pré-determinados (numerados de 1.a 5), com treinamento presencial, reduziram suas taxas de 25,9%, em 2009, para 18,3%, em 2010, com diferença significativa (p < 0,001) ( Tabela 1 e Figura 1 ). Os cinco hospitais que não passaram por treinamento (numerados de 1 a 6) não mostraram diferenças nas taxas de mortalidade associadas ao IAMCSST: de 17,8%, em 2009, a 21,2%, em 2010 (p=0,138) ( Tabela 2 e Figura 2 ). Após a conclusão do período de treinamento, as taxas de mortalidade associadas ao IAMCSST em todos os hospitais públicos de São Paulo caíram de 14,31%, em 2009 (julho a dezembro), para 11,25% em 2013 (janeiro a julho) (p < 0,0001, Tabela 3 ).

**Tabela 1:** – Frequência, número de mortes e taxa de mortalidade de 2008-09 a 2010 em cinco hospitais com treinamento

	Frequência (N)		Número de Mortes (N)		Taxa de mortalidade (%)		Valor de p
Hospital	2008-09	2010	2008-09	2010	2008-09	2010	
1	112	127	26	17	23,2	13,4	
2	121	138	26	25	21,5	18,1	
3	142	157	42	28	29,6	17,8	
4	185	189	50	28	27,0	14,8	
5	165	174	44	46	26,7	26,4	
Total	725	785	188	144	25,9	18,3	< 0,001

**Tabela 2:** – Frequência, número de mortes e taxa de mortalidade de 2008-09 a 2010 em cinco hospitais com treinamento

	Frequência (N)		Número de Mortes (N)		Taxa de Mortalidade (%)		Valor de p
Hospital	2008-09	2010	2008-09	2010	2008-09	2010	
1	112	151	19	30	13,9	19,9	
2	214	172	34	28	15,9	16,3	
3	107	147	28	48	26,2	32,7	
4	73	86	16	23	21,9	26,7	
5	198	194	33	30	16,7	15,5	
Total	729	750	130	159	17,8	21,2	0,138

**Tabela 3:** – Frequência, número de mortes e taxa de mortalidade de 2010 a 2013 em todas as unidades de saúde da cidade de São Paulo

Faixa etária (anos)	Frequência (N)		Número de Mortes (N)		Taxa de Mortalidade (%)		Valor de p
	2010	2013	2010	2013	2010	2013	
até 39	290	275	20	21	6,9	7,64	
40-49	909	1.046	70	56	7,70	5,35	
50-59	1.955	2.252	183	141	9,36	6,26	
60-69	1.900	2.297	229	231	12,05	10,06	
70-79	1.384	1.436	280	235	20,23	16,36	
80+	705	738	240	221	34,04	29,95	
Total	7.143	8.044	1.022	905	14,31	11,25	< 0,0001

No período dos três anos, o número de infartos do miocárdio diagnosticados aumentou em 12,61%. Porém, o número de mortes caiu em 177, uma redução absoluta de 3,06%, e uma redução de risco relativo de 21,39%.

Dados do hospital monitorado mostram que: este hospital (número 1 da Figura 1 ) tinha taxa de mortalidade de 23,7% antes do treinamento, e até tinha estreptoquinase não utilizada. Quatro meses depois do início do treinamento, as taxas de mortalidade caíram para 13,9%. Depois do programa educacional, a administração da tenecteplase foi iniciada nos hospitais treinados, e as taxas de mortalidade passaram a cair progressivamente, atingindo 6,7% neste hospital monitorado em 2015.

## Discussão

Embora as taxas de mortalidade reduzidas observadas após o treinamento possam ter sido influenciadas por outros fatores, como mudanças climáticas ou a vacinação contra o vírus influenza, sentimos que não é o caso. A temperatura média foi maior em 2013 do que em 2010, principalmente no inverno (6% maior).^15^ Anteriormente, tínhamos encontrado uma influência da temperatura no número de mortes associadas ao IAM na cidade de São Paulo, mostrando maior associação entre temperaturas mais baixas e maiores taxas de mortalidade. Em comparação a uma temperatura média em 24 horas de 22,6°C, uma média de 13,7°C aumentou o número de mortes em 32,8%. Por outro lado, observamos um aumento de 11,8% nas taxas de mortalidade quando a temperatura subiu de 21,6-22,6°C a 23,8-27,3°C.^16^ A temperatura em São Paulo aumentou 6% em 2013, em comparação a 2010, e isso não pode explicar a redução nas taxas de mortalidade observadas neste estudo, já que um aumento ficou mais evidente no verão – o que deveria fazer subir – e não cair – o número de mortes de acordo com nossos dados. Na verdade, houve um aumento pequeno no número de casos de IAM em 2014,^15^ mas com menores taxas de mortalidade. Além disso, o nível de umidade foi muito similar nesses dois períodos, então as diferenças relacionadas a este fator podem não ter influenciado a redução das taxas de mortalidade. Outro fator que pode ter impactado os resultados é a vacina contra a influenza; porém, ela está disponível desde 1998, e as taxas de vacinação têm sido estáveis, >70%, desde 2000.^16^ Assim, a redução esperada de casos de IAM devido à vacinação já teria ocorrido de 1996 a 2006.^17^ O outro fator de confusão seria um aumento no número de procedimentos de angioplastia primária realizados em São Paulo de 2010 a 2013, mas este não é o caso, de acordo com o Registro Nacional de Intervenções^18^ em hospitais públicos. Na verdade, o oposto foi verdadeiro, porque o número de procedimentos de angioplastia primária caiu de 503, em 2010, para 185, em 2013. Com base em todos esses dados, acreditamos que a extrema redução das taxas de mortalidade observada neste estudo se deve ao programa de treinamento, que começou em maio de 2010.

A porcentagem de mortes causada por IAM ficou estável de 2002 a 2009.^5^ Este número foi muito alto (14%) em comparação com dados dos Estados Unidos (5,9%).^13^ O aumento absoluto no número de casos de IAM de 2010 a 2013 está de acordo com as projeções da Organização Mundial da Saúde,^1^ e deve crescer ainda mais nos próximos vinte anos. Neste estudo, o treinamento básico para equipes de emergência gerou uma redução significativa no número de mortes causadas por IAM (1.022 vs 905), com redução absoluta de 3,06%. Na verdade, o programa abordou duas grandes questões no programa de treinamento: dificuldade de interpretar um eletrocardiograma; e medo de prescrever a terapia trombolítica devido à possibilidade de sangramento cerebral. Sabemos que esses resultados devem ser mantidos, e consideramos que isso só será possível com um programa de treinamento contínuo direcionado a equipes da emergência. Reconhecemos a possibilidade de que a estratégia de tratar o IAM com tenecteplase, seguida da transferência para um hospital de referência, possa ter contribuído com a redução observada nas taxas de mortalidade. Porém, como tínhamos pré-estabelecido o monitoramento em um hospital, foi possível observar uma redução de mortalidade impressionante: de 23,7% para 13,9%, até antes do início da estratégia com tenecteplase e apenas quatro meses após o início do treinamento. Os hospitais que não tiveram o treinamento não demostraram diferenças neste período, e alguns deles apresentaram maiores taxas de mortalidade. Infelizmente, as taxas de mortalidade continuam a crescer entre pacientes com IAMCSST em nossa cidade, e os esforços para mudar este cenário deveriam considerar a estratégia de treinar equipes de emergência e apoiá-los por meio da tele-ecocardiografia e da telemedicina.

### Limitações do estudo

O estudo teve algumas limitações. Não foi possível obter todos os dados que gostaríamos, como número exato de profissionais treinados, número de profissionais que refizeram o treinamento, o início dos sintomas, os tempos de entrada do paciente e porta-balão, assim como tempo de porta-trombólise. Os dados são de alguns anos atrás, mas hoje em dia hospitais públicos que são similares a este do estudo ainda não possuem uma rotina de realiar a trombólise ou de imediatamente transferir esses pacientes para hospitais com capacidade de ICP.

## Conclusão

Concluímos que o treinamento de equipes de emergência reduziu de forma significativa as taxas de mortalidade e morbidade hospitalar causadas por IAM. A estratégia de implementar treinamento e reciclagem no treinamento de pessoal nos hospitais públicos pode salvar vidas.
