# Supplementary material for: Treinamento de Não-Cardiologistas pode Melhorar os Resultados do Tratamento de Infarto Agudo do Miocárdio com Supra de ST
Source: Arq Bras Cardiol. 2021 Aug 2;117(6):1073–8. [Article in Portuguese] doi: 10.36660/abc.20200180 (PMC8757147; doi:10.36660/abc.20200180)
Supplement: Translation

# Translation: Training Non-Cardiologists Could Improve the Treatment Results of ST Elevation Myocardial Infarction

**Authors:** Luiz Antonio Machado Cesar, Antonio Padua Mansur, Rui Fernando Ramos, Carlos Magalhães, João Fernando Monteiro Ferreira, Naide Aparecida de Oliveira, Amaury Zatorre Amaral, Antonio Célio Camargo Moreno

**Affiliations:** 1 Brasil InCor - Instituto do Coração do Hospital das Clínicas da FMUSP, São Paulo, SP - Brasil; 2 Brasil Instituto Dante Pazzanese de Cardiologia, São Paulo, SP - Brasil; 3 Brasil Universidade Nove de Julho, São Paulo, SP - Brasil; 4 Brasil Secretaria da Saúde do Estado de São Paulo, São Paulo, SP - Brasil; 5 Brasil Secretaria Municipal da Saúde, São Paulo, SP - Brasil; 6 Brasil Secretaria de Gestão Pública, São Paulo, SP - Brasil

**Keywords:** Acute Coronary,Syndrome, Myocardial Infarction/drug therapy, Training, Epidemiology, Mortality, Emergency Services

## Abstract

**Background:**

According to the World Health Organization, emerging countries will have an enormous growth in the number of heart attacks and related deaths. The main medical issue in Brazil is mortality caused by acute ST elevation myocardial infarction (STEMI). The Society of Cardiology in the State of São Paulo has never trained non-cardiologists as emergency personnel. Patients usually seek help from emergency departments instead of calling for an ambulance.

**Objectives:**

We aimed at reducing in-hospital death rates from acute myocardial infarction by training emergency personnel in the city of Sao Paulo.

**Methods:**

We used a training program for the personnel of five hospitals with >100 patients admitted with STEMI per year, and at least 15% in-hospital STEMI-associated mortality rate. We performed internet training, biannual-quarterly symposia for up to 400 participants, informative folders and handouts. Statistical analysis used the two proportion comparison test with p <0.05.

**Results:**

Nearly 200 physicians and 350 nurses attended at least one training from May 2010 to December 2013. Initially, many emergency physicians could not recognize an acute myocardial infarction on the electrocardiogram, but tele-electrocardiography is used in some emergency departments to determine the diagnosis. The death rate in the five hospitals decreased from 25.6%, in 2009, to 18.2%, in 2010 (p=0.005). After the entire period of training, the STEMI-associated death rate in all public hospitals of São Paulo decreased from 14.31%, in 2009, to 11.25%, in 2014 (p<0.0001).

**Conclusion:**

Even simple training programs for emergency personnel can greatly reduce acute myocardial infarction death rates in undeveloped countries.

## Introduction

Cardiovascular disease continues to be the leading cause of death in many countries. According to the World Health Organization, emerging countries will present an enormous growth in the number of heart attacks and, consequently, in the number of deaths.^1^ In 2010, Brazil, which had 200 million inhabitants, had an estimated incidence of 116 heart attacks per 100,000 people,^2^ compared to 294 per 100,000 in the United States.^3^ The main medical issue in Brazil is mortality caused by ST elevation myocardial infarction (STEMI), which is no longer the case for developed countries.^4^ The biggest city in Brazil, the São Paulo metropolitan area, has nearly 18 million inhabitants, most of whom depend on the city’s public health system. Public authorities estimate that ≥70% of the population uses public health services, from primary care to specialized treatments. The city’s in-hospital mortality rate due to STEMI in 2009 was 14.1%,^5^ nearly twice as much as the percentage in developed countries. At that time, the available treatment for STEMI in most of the hospitals in São Paulo was thrombolytic therapy with streptokinase. Currently, the public health system has 46 general hospitals, 139 emergency units for basic emergency care, and 400 ambulatory units. Out of the 46 general hospitals, only 6 can provide primary percutaneous coronary intervention (PCI), and there is no organized system to transfer patients to these hospitals for PCI nor to submit patients to coronary angiography immediately after thrombolysis. The Society of Cardiology in the State of São Paulo, which is part of the Brazilian Society of Cardiology, thought to improve the care addressed to these patients. The Society has trained cardiologists for many years, but cardiologists are not in the emergency rooms; instead, internal medicine physicians and specialists such as gynecologists and orthopedists compose the staff of the emergency units.

Training, retraining, and revising are important tools for any medical update, but this may not be necessary for very basic training. Many similar initiatives have brought clear results in other countries.^6 - 12^ Even considering the relative simplicity of diagnosing and treating STEMI, it is uncertain whether or not basic training can substantially reduce mortality rates. In the public hospitals of São Paulo, only those with specialized cardiology units can provide PCI, and STEMI-associated mortality is acceptable (6 to 7%) in these hospitals. Some of the 24 hospitals unable to provide interventional cardiology treatment had a >15% STEMI-associated death rate in 2009, according to data from the Health Secretariat of the State of São Paulo.^13^ The Society of Cardiology in the State of São Paulo has trained cardiologists, including for the diagnosis and treatment of STEMI, since 1976, but has never trained non-cardiologist emergency personnel. The residents of São Paulo do not usually call 193 (similar to 911 in the United States) for acute chest pain, but instead go to emergency rooms, basic health units, and general hospitals in the city. Therefore, we aimed at reducing in-hospital STEMI-associated mortality rates by providing a training program to emergency personnel (physicians, nurses and other staff) in the city of São Paulo.

## Methods

After three meetings with the heads of emergency units and hospital emergency departments, the Society of Cardiology, together with the Secretariat of Health in the State of São Paulo and the Secretariat of Health in the city of São Paulo, designed a training program.

We pre-determined a first target: to teach personnel in the five hospitals with more than 100 STEMI patients per year and ≥15% in-hospital STEMI-associated mortality rate. For these five hospitals, we performed on-site training on Saturdays. During the meetings, we observed that many participants feared starting thrombolytic therapy.

Besides on-site training, we used internet tools for online training, had update meetings, such as symposia for up to 400 participants 2-4 times each year, created informative online folders and handouts. On-site meetings and the symposia included a four-hour training program with three seminars discussing differential diagnosis of chest pain, thoracic pain, and diagnosis and treatment of acute myocardial infarction (AMI) in the emergency department. After each training, a Q&A session was held. Physicians, nurses and other emergency personnel were invited.

The main goal was to evaluate the effects of the training on in-hospital STEMI-associated mortality rates. During this three-year period, streptokinase was replaced with tenecteplase in some hospitals. After treatment with the thrombolytic, aspirin, clopidogrel, and enoxaparin, patients were transferred to a tertiary hospital capable of providing interventional cardiology treatment and surgery. The incidence of AMI and associated death rate was updated every semester by the Secretariat of Health in the State of São Paulo. This information came from public hospitals, which filled out forms including admission, discharge and in-hospital mortality rates.

To evaluate the influence of both the training program and the strategy of tecnecteplase followed by transfer to a reference hospital on mortality rates, we specifically monitored one hospital in which tecnecteplase was introduced and evaluated mortality rates after four months of training, and then after the onset of the tenecteplase strategy along the years, after 2013 until 2015. Besides, we took the same data from five other hospitals with ≥15% in-hospital STEMI-associated mortality rate as a control. An Institutional Review Board (IRB) approved this study, whose data was collected from the Secretariat of Health in the State of São Paulo, Brazil.

### Statistical Analysis

For this study, we obtained data from the Secretariat of Health in the city of São Paulo. The database contained data from each health unit where a patient had been admitted by generating an authorization for hospitalization. We used number of deaths (N) and mortality rates (%) to perform the two proportion comparison test, using the statistic software Primer of Biostatistics^®^, version 4.02.9.^14^ P-value was significant when < 0.05.

## Results

We provided on-site training to the emergency personnel in five hospitals and extended the training to three other hospitals in 2010. The same participants were retrained in online meetings and update symposia. Twelve emergency units also trained their staff. Many other hospitals trained their emergency and intensive care unit personnel from 2011 to 2013. In total, nearly 200 physicians and 350 nurses attended at least one training session from May, 2010, to December, 2013. We observed that many emergency physicians were unable to identify an AMI on the electrocardiogram. Nearly 50 emergency departments used tele-electrocardiography, causing a five-minute delay in diagnosis. Even in those departments, the emergency staff often feared starting thrombolytic therapy. The STEMI-associated death rates in the five pre-determined hospitals (numbered from 1 to 5) with on-site training reduced their rates from 25.9%, in 2009, to 18.3%, in 2010, with significant difference (p < 0.001) ( Table 1 and Figure 1 ). The five non-trained hospitals (numbered from 6 to 10) did not show differences in STEMI-associated death rates: from 17.8%, in 2009, to 21.2%, in 2010 (p=0.138) ( Table 2 and Figure 2 ). After the entire training period, the in-hospital STEMI-associated death rates in all the public hospitals of São Paulo decreased from 14.31%, in 2009 (July-December), to 11.25%, in 2013 (January-July) (p < 0.0001, Table 3 ).

**Table 1:** – Frequency, number of deaths and mortality rate from 2008-09 to 2010 in the five trained hospitals

	Frequency (N)		Number of Deaths (N)		Mortality rate (%)		p-value
Hospital	2008-09	2010	2008-09	2010	2008-09	2010	
1	112	127	26	17	23.2	13.4	
2	121	138	26	25	21.5	18.1	
3	142	157	42	28	29.6	17.8	
4	185	189	50	28	27.0	14.8	
5	165	174	44	46	26.7	26.4	
Total	725	785	188	144	25.9	18.3	< 0.001

**Figure 1:** – Mortality rate (%) in the first five hospitals attending the first training program. Comparison between 2008-2009 and 2010 (p-value for comparison). Columns: gray= before; and dash= after training. The numbers on the columns show the exact percentage death rate in each hospital.

**Table 2:** – Frequency, number of deaths and mortality rate from 2008-09 to 2010 in the five trained hospitals

	Frequency (N)		Number of Deaths (N)		Mortality rate (%)		p-value
Hospital	2008-09	2010	2008-09	2010	2008-09	2010	
1	112	151	19	30	13.9	19.9	
2	214	172	34	28	15.9	16.3	
3	107	147	28	48	26.2	32.7	
4	73	86	16	23	21.9	26,7	
5	198	194	33	30	16.7	15.5	
Total	729	750	130	159	17.8	21.2	0.138

**Figure 2:** – Mortality rate (%) in the five hospitals that did not receive the training program. Comparison between 2008-2009 and 2010 (p-value for comparison). Columns: gray= before; and dash= after. The number on the columns show the exact percentage death rate in each hospital.

**Table 3:** – Frequency, number of deaths and mortality rate from 2010 to 2013 in all health units in the city of São Paulo

Age range (years)	Frequency (N)		Number of Deaths (N)		Mortality rate (%)		p-value
	2010	2013	2010	2013	2010	2013	
up to 39	290	275	20	21	6.9	7.64	
40-49	909	1,046	70	56	7.70	5.35	
50-59	1,955	2,252	183	141	9.36	6.26	
60-69	1,900	2,297	229	231	12.05	10.06	
70-79	1,384	1,436	280	235	20.23	16.36	
80and+	705	738	240	221	34.04	29.95	
Total	7,143	8,044	1,022	905	14.31	11.25	< 0.0001

Over the three-year period, the number of diagnosed myocardial infarctions increased by 12.61%. However, the number of deaths decreased by 177, an absolute reduction of 3.06% and a relative risk reduction of 21.39%.

Data from the monitored hospital were as follows: this hospital (number 1 in Figure 1 ) had a 23.7% mortality rate before training, and even had streptokinase that was not used. Four months after the beginning of the training, mortality rates decreased to 13.9%. After the educational program, the administration of tenecteplase was started in trained hospitals and mortality rates progressively decreased, reaching 6.7% in this monitored hospital in 2015.

## Discussion

Although the reduced mortality rates we observed after training could have been affected by other factors, such as weather changes or *H. influenza* vaccination, we feel this is not the case. The median temperature was higher in 2013 than in 2010, especially in the winter, being 6% higher.^15^ We had previously found an influence of temperature on the number of AMI-associated deaths in the city of São Paulo, showing a strong association between lower temperatures and increasing death rates. Compared to an average 24-hour day temperature of 22.6^o^C, an average of 13.7^o^C increased the number of deaths in 32.8%. On the other hand, we observed an increase of 11.8% in death rates when temperatures rose from 21.6-22.6ºC to 23.8-27.3^o^C.^16^ The temperature in São Paulo had a 6% increase in 2013, in comparison to 2010, and this could not explain the reduction in death rates observed in this study, since such an increase was more evident in the summer, which should rise – and not reduce – the number of deaths according to our data. In fact, there was a very slight growth in the number of cases of AMI in 2014,^15^ but with lower death rates. In addition, the level of humidity was very similar between these two periods, so differences related to this factor could not have influenced the reduction of death rates. Another factor that could have had an impact on the results is the vaccine for the influenza virus; however, it has been available since 1998, and vaccination rates have been stable, >70%, since 2000.^16^ Therefore, the expected reduction in cases of AMI due to vaccination had already occurred as of 1996-2006.^17^ The other confounding factor would be an increase in the number of primary angioplasty procedures performed in São Paulo from 2010 to 2013, but this is not the case, according to the National Registry of Interventions^18^ in public hospitals. Actually, the opposite was true, because the number of primary angioplasty procedures decreased from 503, in 2010, to 185, in 2013. Based on all of these data, we believe that the extreme reduction in mortality rates observed in this study was owed to the training program, which started in May, 2010.

The percentage of deaths caused by AMI remained stable from 2002 to 2009.^5^ This number was very high (14%), compared to the data in the United States (5.9%).^13^ The absolute increase in the number of cases of AMI from 2010 to 2013 is in accordance with the projections from the World Health Organization,^1^ and is supposed to grow even more in the next twenty years. In this study, basic training of emergency personnel caused a significant reduction in the number of deaths due to AMI (1,022 vs 905), with an absolute reduction of 3.06%. In fact, there were two main issues addressed by the training program: difficulty in interpreting an electrocardiogram; and fear of prescribing thrombolytic therapy due to the possibility of brain bleed. We know that these achieved results must be maintained, and we consider this will only be possible with a continuous training program directed to emergency personnel. We acknowledge the possibility that the strategy of treating AMI with tenecteplase, followed by transfer to a reference hospital, could have contributed with the observed reduction in mortality rates. However, as we had pre-established the monitoring of one hospital, it was possible to observe an impressive mortality reduction: from 23.7% to 13.9%, even before the beginning of the strategy with tenecteplase and just four months after the training had started. The non-trained hospitals did not show any differences in this period, and some of them even presented higher mortality rates instead. Unfortunately, mortality rates continue to rise among STEMI patients in our city, and efforts to change this scenario should strongly consider the strategy of training emergency personnel and giving support through tele- electrocardiography and telemedicine.

### Study limitations

The study had some limitations. It was not possible to have all the data we wanted, such as the exact number of trained professionals, the number of retrained professionals, the onset of symptoms, the onset-to-door and door-to-balloon times, as well as door-to-thrombolysis time. The data were from a few years ago, but nowadays public hospitals that are similar to that in this study still do not have a routine to carry out thrombolysis or immediate transfer to hospitals that are capable of PCI.

## Conclusion

In conclusion, the training of emergency personnel significantly reduced AMI in-hospital morbidity and mortality rates. The strategy of implementing personnel training and retraining in public hospitals is life-saving.
